# BRICK-Automated Virtual Temperature Sensors for Sensor Fault Detection, Isolation, and Discrimination in Smart-Building HVAC Systems

**DOI:** 10.3390/s26113465

**Published:** 2026-05-31

**Authors:** Khaled Chahine, Hassan N. Noura

**Affiliations:** 1 College of Engineering and Technology, American University of the Middle East, Egaila 54200, Kuwait; khaled.chahine@aum.edu.kw; 2 Electrical and Computer Engineering Department, American University of Beirut, Beirut 1107 2020, Lebanon; 3 Institut FEMTO-ST, CNRS, IUT-NFC, Université Marie et Louis Pasteur, F-90000 Belfort, France

**Keywords:** sensor fault detection, BRICK schema, virtual sensor, HVAC, fault isolation, fault discrimination, LightGBM, smart buildings, building metadata, inter-sensor consistency

## Abstract

Sensor bias faults in closed-loop HVAC systems pose a detection challenge that is both subtle and costly. Because the control loop compensates for biased readings by driving the affected sensor back toward its setpoint, the fault becomes invisible to conventional threshold monitors. The anomaly does not vanish, however; it is redistributed across correlated sensors, disrupting their mutual consistency. We propose a framework that automatically derives virtual temperature sensor models from BRICK schema metadata. LightGBM regressors, trained on fault-free inter-sensor relationships, produce z-scored prediction residuals that serve as detection signals. Fault isolation is achieved by ranking sensors by their median daily anomaly scores; fault-type discrimination relies on analysis of actuator command-position discrepancies. On the Lawrence Berkeley National Laboratory (LBNL) fault detection and diagnosis (FDD) benchmark, the method achieves an area under the receiver operating characteristic curve (AUC) of 0.9992 for the mildest sensor bias (SA +2 °C), an AUC of 1.0 for all other single-duct air handling unit (SD-AHU) scenarios, and an AUC of 1.0 for all fan coil unit (FCU) sensor bias scenarios. In all four SD-AHU sensor bias scenarios, the biased sensor (SA_TEMP) ranks first or second; for the larger biases (±4 °C), SA_TEMP consistently ranks first. A robustness analysis over 10 random seeds confirms that detection AUC remains above 0.997 in all cases. Sensor and mechanical faults fall into non-overlapping clusters in the command-position discrepancy space. On the FCU system, the proposed method substantially outperforms principal component analysis (PCA) (AUC = 1.0 versus 0.63–0.90) and provides diagnostic capabilities not available with PCA. Notably, a single pipeline function handles both system types without modification, confirming cross-system scalability through the BRICK metadata layer. The results confirm that BRICK-automated virtual sensor construction is a viable approach for scalable, deployment-ready sensor validation in smart-building HVAC systems.

## 1. Introduction

Buildings account for approximately 30–40% of global final energy consumption, with heating, ventilation, and air-conditioning (HVAC) systems being responsible for nearly half of a building’s energy use [1,2]. A significant portion of this energy is wasted due to degraded equipment and undetected sensor faults rather than poor design. Katipamula and Brambley estimate that HVAC faults account for 15–30% of energy waste in commercial buildings [1]. Sensor bias faults are the most insidious of these: they produce no alarms, yet they quietly distort the control inputs that govern damper positions, valve commands, and chiller staging.

Why sensor bias is so difficult to detect in closed-loop HVAC systems stems from a phenomenon that the FDD literature has largely overlooked. When a temperature sensor drifts, the proportional–integral–derivative (PID) controller responds by adjusting valve openings, fan speeds, and damper positions to maintain the apparent setpoint. Consequently, the biased sensor’s own time series reverts to near-normal values, and any monitor that examines the sensor in isolation sees nothing faulty. The fault signature migrates elsewhere: the control system, working harder to compensate, drives correlated sensors away from their expected trajectories. Granderson et al. explicitly documented this mechanism: when a +2 °C zone temperature bias was injected into the simulation, the anomaly appeared in the cooling coil valve position rather than in the temperature sensor itself [3]. Kim and Katipamula reported a similar finding: the feedback structure of closed-loop control masks sensor fault symptoms, causing PCA-based methods to fail on this fault category [2].

Current FDD approaches have limited effectiveness in addressing sensor faults. Rule-based methods, such as the Air Handling Unit Performance Assessment Rules (APAR), encode mass-balance and energy-conservation principles into 28 heuristic rules [4]. APAR can flag certain sensor deviations, but it must be manually configured for every new AHU and offers no fault isolation [4]. Physics-based residuals, such as the mixing box energy balance, are ineffective at detecting sensor biases that do not directly enter the balance equation; the biased temperature sensor lies downstream of the mixing point and is not a variable in the energy balance, while the controller’s compensatory damper adjustments shift both sides of the equation approximately together, leaving the residual non-discriminative. We confirm this quantitatively in Section 3.1. PCA with wavelet preprocessing can detect anomalies through global reconstruction error [5]. Li and Wen reported good results for abrupt mechanical faults, but PCA only detects; it cannot isolate the faulty sensor, and it struggles with gradual degradation [5]. Wang et al. extended PCA-based detection to a system-level strategy for central chilling plants, using Q-contribution analysis to isolate the specific faulty sensor and achieving detection ratios above 80% on simulated data [6]. Visek et al. applied PCA and fuzzy-PCA to reconstruct temperature sensor signals in vapor compression systems, enabling fault detection through residual analysis and continued system operation via signal replacement [7]. Generative adversarial networks tackle data imbalance in fault classification [8], but like all supervised methods, they require labeled fault data, a resource that is scarce in operational buildings [9,10]. Yan et al. proposed a Hidden Markov Model framework for AHU component and sensor fault diagnosis that can detect both known and previously unmodeled fault types by dynamically updating the model using Bayes-factor hypothesis testing; the method achieved F-measures above 0.99 for sensor bias and drift on simulated data and outperformed SVM-based classifiers on ASHRAE benchmark data [11]. Gunay et al. used inverse greybox modeling to detect zone-level sensor and actuator faults in a real building without requiring labeled fault data, but noted that the lack of standard metadata models such as BRICK and Haystack makes mapping BAS data points a labor-intensive barrier to deploying such methods across large building portfolios [12].

A significant barrier to practical FDD deployment is scalability across diverse buildings. Each building features unique sensor naming conventions, equipment topologies, and system configurations. As a result, algorithms developed for one installation require substantial re-engineering before they can be applied elsewhere [1,10]. The BRICK metadata schema tackles this heterogeneity through a standardized ontology of building entities, types, and relationships [13]. Three foundational relationship types (hasPoint, hasPart, and feeds) connect sensors to equipment and equipment to downstream zones. Validation across six commercial buildings, encompassing over 17,700 data points from five building management system (BMS) vendors, demonstrated that BRICK successfully mapped 98% of sensor metadata and enabled eight portable analytics applications, including FDD, to operate across all buildings without modification [13]. The Mortar open testbed, which uses BRICK as its metadata foundation, extends this portability to 90 buildings and over 9.1 billion data points, demonstrating that BRICK-based applications can scale to portfolio-level analytics [14]. Recent extensions to BRICK, such as fault–symptom relationships (FSBrick), show that 88.2% of fault types and 87.7% of fault severities documented in the FDD literature can be formally represented, enabling automated revision of diagnostic models when building configurations change [15]. Papadopoulos et al. proposed a distributed model-based architecture in which each building zone is monitored by a local diagnostic agent; new zones can be added via a plug-and-play mechanism without redesigning the global system, demonstrating scalability to an 83-zone building [16]. However, this approach requires explicit physical models for each subsystem and does not leverage building metadata standards. To the authors’ knowledge, however, no prior work has used BRICK metadata to automate the construction of the FDD model itself, that is, to define which sensors should predict which others.

Virtual sensors, which are data-driven models that predict a physical sensor’s value from correlated measurements, provide an effective mechanism for detecting sensor faults: a fault is indicated when the physical reading diverges from its predicted value. Bellanco et al. reviewed virtual-sensor applications for heat pump FDD and concluded that virtual sensors are essential for reducing instrumentation costs, but have been applied in only a limited number of FDD studies to date [17]. Gao et al. (2019) applied LSTM-based virtual sensors to chiller sensor fault diagnosis, using the maximal information coefficient to select correlated sensor groups, and reported strong per-sensor fault detection [18]. Liu et al. combined virtual sensing with Bayesian inference and Markov chain Monte Carlo sampling to achieve self-calibration accuracies exceeding 98% on air handling unit (AHU) temperature and component faults [19]. Yoon coupled an autoencoder with Bayesian inference for in situ AHU sensor calibration, achieving residual errors within ±0.2 °C after correcting a +2 °C temperature bias on simulated EnergyPlus data [20]. Choi and Yoon extended this approach using virtual sensors constructed via Gaussian process regression, reducing the calibration error rate from 72.7% to 5.2% when multiple simultaneous sensor errors were present [21]. Elnour et al. demonstrated that an auto-associative neural network can isolate individual sensor faults through per-sensor reconstruction residuals on a simulated two-zone HVAC system [22]; the extended journal study tested bias, drift, complete failure, and precision degradation faults on a three-zone system, achieving an 85% true-positive rate with 3% false positives [23]. However, each of these approaches requires manual selection of the sensor neighborhood from which virtual models are constructed. Gao et al. explicitly identified cross-system generalization as an open problem [18]. The closest metadata-driven competitor, Gao et al. (2024), automatically derived a fault diagnosis Bayesian network from Project Haystack metadata [24], but focused on building-level mechanical faults and identified sensor bias as a limitation that requires future work. Smagulova and Cerpa’s FADED framework addressed sensor fault classification in variable-air-volume (VAV) units by deploying a parallel physical sensing infrastructure: a technician collects an independent 30-min reference signal using a smartphone app, a wireless anemometer, and a Bluetooth temperature sensor [25]. FADED achieved 99.57% zone temperature classification accuracy on real building data, but required physical-sensor deployment and manual site visits, and did not use standardized building metadata. Hosamo et al. integrated BRICK into a digital twin framework for AHU condition monitoring, but BRICK was used solely for data integration, while the FDD logic was provided by hand-coded APAR rules and machine learning models for maintenance scheduling [26].

This paper presents a framework that bridges the gap between BRICK metadata and the construction of automated FDD models. Three primary contributions are offered. First, a BRICK-automated virtual-sensor derivation pipeline is introduced, in which the metadata schema directly determines, for each temperature sensor, the set of correlated sensors used to train a LightGBM prediction model, eliminating the need for manual sensor selection. Second, the framework is shown to provide detection, isolation, and fault type discrimination: per-sensor anomaly ranking localizes the faulty sensor, and actuator command-position discrepancy analysis separates sensor faults from mechanical faults. Third, the framework is validated on two physically distinct HVAC system types from the LBNL FDD benchmark [3,27], a single-duct variable-air-volume AHU and a four-pipe FCU, using the same pipeline function and without system-specific modifications. For the FCU system, the proposed method substantially outperforms PCA on sensor bias detection, while also providing diagnostic capabilities that PCA cannot offer.

## 2. Materials and Methods

### 2.1. Abbreviations and Notation

We start this section by providing a list of the abbreviations, mathematical notation, and metrics used in the paper. Table 1 is structured into four groups, covering general abbreviations, HVAC-specific variables, mathematical notation, and derived diagnostic metrics.

### 2.2. Dataset Description

All experiments use the LBNL FDD benchmark dataset [3,27], the largest publicly available labeled dataset for building HVAC fault detection. EnergyPlus-Modelica co-simulation produced the SD-AHU data; HVACSIM+ produced the FCU data. Each fault–severity combination spans a full year at one-minute resolution. Chicago TMY weather drives the SD-AHU; Des Moines TMY drives the FCU. Both systems ship with BRICK .ttl schema files. Temperatures appear in degrees Fahrenheit; bias severities (±2 °C, ±4 °C) denote the injected offset in Celsius, following the LBNL convention.

The SD-AHU, a single-duct variable-air-volume air handling unit, records 30 variables: supply, mixed, return, and outdoor air temperatures; supply and return airflows; damper and valve positions and commands; fan speeds and power; and five zone temperatures. Dataset inspection yielded twelve usable scenarios: one fault-free baseline, four supply air temperature (SA_TEMP) sensor bias scenarios (+2 °C, +4 °C, −2 °C, −4 °C), three outdoor air-damper-stuck scenarios (10%, 25%, 75% stuck), three cooling-coil-valve-stuck scenarios (10%, 50%, 75% stuck), and one cooling coil valve leak scenario. The four outdoor-air-temperature bias files turned out to be duplicates of the fault-free baseline (a packaging error), so OA sensor faults are excluded. Each scenario contains 525,540 rows, corresponding to approximately one full calendar year (January through December) at a one-minute temporal resolution; the use of Chicago Typical Meteorological Year weather data ensures that all four seasons are represented. The full scenario inventory is given in Table 2.

**Figure 1:**
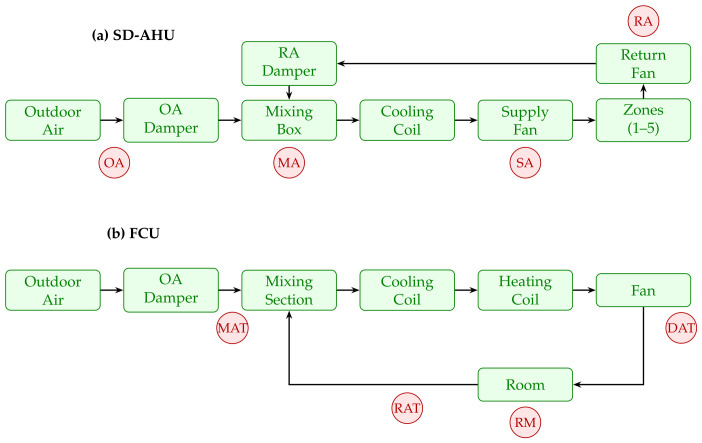
Schematic diagrams of the two HVAC systems investigated. (**a**) The SD-AHU system: outdoor air passes through a damper into the mixing box, where it blends with recirculated return air. The mixed air is conditioned by the cooling coil, driven by the supply fan through five zones, and returned by the return fan. Red circles indicate temperature sensor locations (OA = outdoor air, MA = mixed air, SA = supply air, RA = return air). (**b**) The FCU system: Outdoor air enters through a damper into the mixing section, passes through cooling and heating coils, and is delivered to the room by a fan. Return air from the room recirculates to the mixing section. Temperature sensor locations include MAT = mixed air, DAT = discharge air, RM = room, RAT = return air.

In the FCU system, the four-pipe hydronic FCU includes 29 variables, including room temperature, mixed air temperature, discharge air temperature, return air temperature, chilled-water supply and return temperatures, hot-water supply and return temperatures, and damper or valve positions. Ten scenarios were analyzed: one fault-free baseline, four room temperature (RM_TEMP or FCU_RAT) sensor bias scenarios (±2 °C, ±4 °C), two cooling-valve-stuck scenarios (50% and 100%), one heating-valve-stuck scenario (50%), and two outdoor-air-damper-stuck scenarios (50% and 100%). Each FCU scenario contains 525,600 rows at a one-minute resolution, spanning one full calendar year, driven by Des Moines Typical Meteorological Year weather data, which covers all four seasons. The schematic diagrams of both systems are shown in Figure 1.

### 2.3. BRICK-Automated Target Derivation

**Figure 2:**
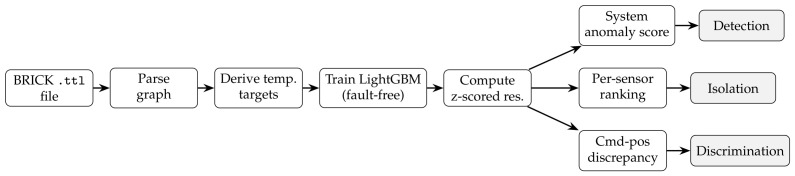
Framework overview. The BRICK schema file is parsed to derive temperature sensor prediction targets. LightGBM models trained on fault-free inter-sensor relationships produce z-scored residuals that enable fault detection, isolation, and discrimination.

The central methodological contribution of this work is the automated derivation of virtual-sensor prediction targets and their associated feature sets from BRICK metadata. Existing metadata-driven FDD approaches use building ontologies for data integration or fault-symptom encoding, but none use the metadata to construct the data-driven prediction model itself. Hosamo et al. use BRICK to map sensor data streams into a digital twin, but the FDD logic consists of hand-coded APAR rules and separate machine learning models for maintenance scheduling [26]. Hwang et al.’s FSBrick extension adds fault–symptom relationships to the BRICK ontology, enabling automated revision of diagnostic models when configurations change, but the diagnostic model itself must still be specified by an expert [15]. Fierro et al.’s Mortar testbed provides a BRICK-based data platform spanning 90 buildings and 9.1 billion data points, but it offers no FDD logic [14]. Gao et al. (2024) automatically derive a Bayesian network from Project Haystack metadata, but their system targets building-level mechanical faults and explicitly identifies sensor bias as a limitation requiring future work [24]. In contrast, the framework presented here uses BRICK graph traversal to determine, for each temperature sensor, which correlated sensors feed its virtual model. The metadata thus configures the FDD model, not merely the data pipeline. Figure 2 provides an overview of the complete pipeline.

Algorithm 1 formalizes this procedure. Each system’s BRICK .ttl file is parsed using rdflib to extract entities, their BRICK class types, and the edges connecting them. Six relationship types are retained: hasPoint, hasPart, feeds, and their inverses (isPointOf, isPartOf, isFedBy).

Three design decisions in Algorithm 1 warrant explanation. First, the exogenous filter in Phase 2 excludes outdoor air temperature automatically because its BRICK class type (Outside_Air_Temperature_Sensor) contains the “Outside” substring. Outdoor air temperature is determined by weather conditions external to the HVAC system and cannot be reliably predicted from building-internal measurements (validation RMSE = 16.88 °F, compared to 0.13–0.99 °F for internal temperature sensors). Second, the minimum feature threshold of three in Phase 3 prevents degenerate models from being constructed for sensors with too few correlated neighbors. Third, the fallback in Phase 4 activates only when the primary BRICK path yields zero matching columns, as occurs in the FCU dataset where BRICK entity names use class identifiers (e.g., Mixed_Air_Temperature_Sensor) rather than CSV column headers (e.g., FCU_MAT). Both code paths reside in the same derive_targets() function.

For each identified target *s*, we append temporal context features to F(s): hour of day, month, day of week, and a single system-level binary occupancy indicator derived from the system operation status signal (SYS_CTL ≥1 indicates occupied mode). These features capture diurnal and seasonal operating patterns.

**Figure 3:**
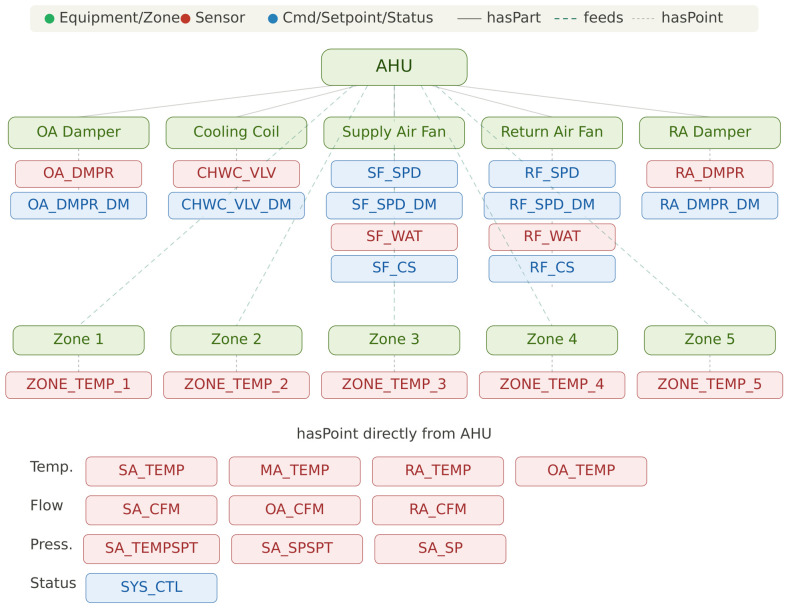
BRICK schema graph for the SD-AHU system. Nodes represent entities colored by type (equipment and zones: green; sensors and measurement points: red; commands and status indicators: blue). Edges encode hasPoint, hasPart, and feeds relationships. The graph topology determines, for each temperature sensor target, the set of features used to train its virtual-sensor model.

The SD-AHU .ttl file enumerates 41 BRICK entities (Table 3), comprising the AHU unit, cooling coil, supply and return fans, outdoor air damper, return air damper, five HVAC zones, and 30 sensor, command, or setpoint points. Eight temperature sensor targets were automatically derived: SA_TEMP, MA_TEMP, RA_TEMP, and ZONE_TEMP_1 through ZONE_TEMP_5. OA_TEMP was excluded automatically by the exogenous filter in Phase 2 because its BRICK class contains the “Outside” substring. The BRICK graph for this system is shown in Figure 3.
**Algorithm 1** BRICK-automated virtual-sensor target derivation.**Require:** BRICK entities *E*, edges *R*, CSV column names *C***Ensure:** Target map *T*: sensor → feature set**  ** **Phase 1: Build equipment-to-sensor map**1:**for** each edge $(e_{i},\;\mathrm{hasPoint},\;s_{j})\in R$ **do**2:    **if** $s_{j}\in C$ **then**3:        $\mathrm{EquipSensors}\left[e_{i}\right]\leftarrow \mathrm{EquipSensors}\left[e_{i}\right]\cup \left\{s_{j}\right\}$4:    **end if**5:**end for**6:**for** each edge $(e_{i},\;\mathrm{hasPart},\;e_{k})\in R$ **do**7:    **for** each edge $(e_{k},\;\mathrm{hasPoint},\;s_{j})\in R$ where $s_{j}\in C$ **do**8:        $\mathrm{EquipSensors}\left[e_{i}\right]\leftarrow \mathrm{EquipSensors}\left[e_{i}\right]\cup \left\{s_{j}\right\}$9:    **end for**10:**end for**** ****Phase 2: Identify temperature sensor targets**11:**for** each equipment $e_{i}$ and sensor $s\in \mathrm{EquipSensors}[e_{i}]$ **do**12:    τ← BRICK class types of *s*13:    **if** “Temperature” ∈τ **and** “Setpoint” ∉τ **and** “Outside” ∉τ **then**14:        Mark *s* as a prediction target under $e_{i}$15:    **end if**16:**end for**** ****Phase 3: Construct feature sets**17:**for** each target *s* under equipment $e_{i}$ **do**18:    $F\left(s\right)\leftarrow \mathrm{EquipSensors}\left[e_{i}\right]\setminus \left\{s\right\}$            ▹ same-equipment sensors19:    **for** each equipment $e_{k}\neq e_{i}$ **do**20:        $F\left(s\right)\leftarrow F\left(s\right)\cup (\mathrm{EquipSensors}\left[e_{k}\right]\cap C)$21:    **end for**22:    **if** |F(s)|≥3 **then**23:        T[s]←F(s)24:    **end if**25:**end for**** ****Phase 4: Column-name fallback**26:**if** T=∅ **then**27:    Identify columns in *C* ending with HVAC temperature suffixes         (_TEMP, _MAT, _DAT, _RAT, _EWT, _RWT),         excluding columns containing SPT (setpoints) or _OA (outdoor air)28:    **for** each such column *t* **do**29:        T[t]←C∖{t}30:    **end for**31:**end if**32:**return** *T*

When we applied the same function to the FCU .ttl file, the primary BRICK path produced zero matching columns because the entity names did not correspond to the CSV headers. The fallback heuristic activated and identified eight temperature columns: FCU_CLG_EWT, FCU_CLG_RWT, FCU_DAT, FCU_HTG_EWT, FCU_HTG_RWT, FCU_MAT, FCU_RAT, and RM_TEMP. This design accommodates naming inconsistencies that are common in real-world BRICK deployments.

### 2.4. Virtual Temperature Sensor Models

For each temperature target *s*, we trained a LightGBM gradient-boosted tree regressor to predict the sensor value from F(s)—the BRICK-derived neighbors plus temporal context. The first 80% of fault-free occupied timesteps (ordered chronologically) formed the training set; the last 20% formed the validation set. This temporal ordering prevents data leakage. Unoccupied hours (SYS_CTL = 0) were excluded because their dynamics differ from the operational regime that the virtual sensor must capture. Occupancy runs approximately 06:00 to 22:00 on weekdays.

We fixed LightGBM hyperparameters across all targets and both systems: 300 estimators, max_depth = 6, learning_rate = 0.05, subsample = 0.8, colsample_bytree = 0.8, random_state = 42, and min_child_samples = 50. No per-system tuning was performed. These values were selected as conservative defaults that balance model capacity against overfitting risk: a moderate depth of 6 with subsampling rates of 0.8 limits tree complexity, while having 50 minimum child samples prevents splits on small data subsets. The fact that the same configuration produces accurate virtual sensors on both the SD-AHU (RMSE = 0.13–0.99 °F across internal temperature targets) and the FCU system, without any system-specific adjustment, provides evidence of robustness to system differences. The temporal split is deliberate: shuffling the training data would leak seasonal patterns from later months into earlier-month predictions, violating the operational assumption that models are trained on a contiguous fault-free commissioning period. Formal cross-validation and confidence intervals were not computed; this is acknowledged as a limitation in Section 4.

On the validation set, we computed the absolute residual $e_{t,s}=\left|y_{t,s}-{\hat{y}}_{t,s}\right|$ at every timestep *t*. The resulting baseline distribution $N(\mu_{s},\sigma_{s}^{2})$ characterizes normal model uncertainty for sensor *s*. Table 4 presents the root mean squared error (RMSE), mean absolute error (MAE), and standard deviation of the absolute residual for each SD-AHU temperature target. Supply air temperature has the lowest error (RMSE = 0.1516, MAE = 0.0388), reflecting its tight deterministic coupling to the supply fan and cooling coil valve. Zone temperatures exhibit larger residuals; ZONE_TEMP_5 reaches an RMSE of 0.9865, which we attribute to occupant-driven variability in that peripheral zone. OA_TEMP is omitted owing to the packaging error noted in Section 2.2.

Training all eight models on the SD-AHU fault-free data (220,000 occupied timesteps after the 80/20 split) took under two min on a single CPU core (Google Colab, Intel Xeon, 2.2 GHz). Scoring one day of 1-min data across all eight models took under one second. These computational requirements are modest: training completes on a single CPU core without GPU acceleration, and inference adds negligible latency to a one-minute sampling loop. In contrast, deep learning alternatives (autoencoders, LSTM networks) typically require GPU resources for training. We note, however, that BMS controller hardware varies widely, and the Google Colab benchmark reported here may not directly translate to all edge platforms.

### 2.5. Anomaly Detection

We define the z-scored absolute residual for sensor *s* at timestep *t* as

(1) $$z_{t,s}=\frac{|y_{t,s}-{\hat{y}}_{t,s}|-\mu_{s}}{\sigma_{s}}$$

where $|y_{t,s}-{\hat{y}}_{t,s}|$ denotes the absolute prediction residual, and $\mu_{s}$ and $\sigma_{s}$ are the mean and standard deviation of the absolute residual distribution computed on the fault-free validation set. Note that both the numerator and the baseline statistics operate on absolute residuals, not signed residuals. We aggregate daily by averaging $z_{t,s}$ over occupied timesteps in each calendar day, yielding a per-sensor daily score $Z_{d,s}$. The system-level score is the maximum: $Z_{d}=\max_{s}Z_{d,s}$.

We set a detection threshold at z=3 (approximately the 99.7th percentile under a Gaussian assumption): a day is faulty when $Z_{d}>3$. The system-level score $Z_{d}\;=\;\max_{s}Z_{d,s}$ takes the maximum across all per-sensor daily scores, meaning that a single sensor exceeding the threshold is sufficient to flag the day as anomalous. This max-aggregation ensures that a localized fault affecting one sensor is not averaged away by the healthy scores of the remaining sensors. The z=3 threshold follows standard statistical process control practice; its effect on detection performance across different thresholds is examined later. ROC evaluation uses only the last 20% of the time series (the validation period) to prevent data leakage. Fault-free validation days form the negative class; the same dates from each fault scenario form the positive class.

### 2.6. Fault Isolation

Fault isolation exploits the z-score ranking structure. For each fault scenario, we ranked sensors by their median daily anomaly score ${\tilde{Z}}_{s}$, computed over the full fault-scenario period. The median was chosen over the mean because it is robust to outlier days, such as those at seasonal transitions or during atypical operating conditions, that can produce spurious spikes in individual daily scores. The top-ranked sensor is the most probable fault location. The gap between rank-1 and rank-2 scores, ${\tilde{Z}}_{\left(1\right)}-{\tilde{Z}}_{\left(2\right)}$, serves as an isolation margin: a large gap signals unambiguous localization. No formal threshold is set on the isolation margin; instead, we report the numerical margins for each scenario in Section 3.3 so that the reader can assess localization confidence.

### 2.7. Fault Type Discrimination

Two command-position discrepancy signals were calculated using the actuator channels provided in the SD-AHU dataset:

- Damper_MAE = MAE(OA_DMPR_DM, OA_DMPR)
- Valve_MAE = MAE(CHWC_VLV_DM, CHWC_VLV)

In the presence of sensor bias faults, actuators continue to respond accurately to their commands, keeping both MAEs near zero. In contrast, mechanical faults cause the physically impaired actuator to exhibit a significantly elevated MAE. When these two MAEs are combined with the system-level anomaly score, the resulting three-dimensional discrimination space is used to assess whether sensor, damper, and valve faults can be separated. The clustering behavior observed in this space is reported in Section 3.4.

### 2.8. Baseline Methods

We implemented two baseline detectors on the same 80/20 temporal split.

*Mixing box energy balance residual.* The mixing box equation estimates mixed air temperature as a damper-fraction-weighted blend of outdoor and return air temperatures:

(2) $${\mathrm{MA}\_\mathrm{TEMP}}_{\mathrm{pred}}=\alpha \cdot \mathrm{OA}\_\mathrm{TEMP}+\left(1-\alpha \right)\cdot \mathrm{RA}\_\mathrm{TEMP}$$

where α=OA_DMPR, representing the outdoor air damper fraction, which ranges from 0 to 1. This formulation assumes a linear relationship between damper position and outdoor air fraction, which is a simplification; real damper characteristics are typically nonlinear. However, this is the standard formulation used by the APAR rules [4], and, as shown in Section 3.1, the mixing box residual fails to detect sensor bias regardless of this assumption. The daily mean absolute deviation from this predicted value is used as the detection score.

*PCA reconstruction error.* All numeric columns were standardized to zero mean and unit variance using statistics computed from the fault-free 80% training split. We retained PCA components explaining 95% of the variance. The mean squared reconstruction error per day is the detection score. This baseline instantiates the global-statistical-anomaly family reviewed by Li and Wen [5] and implemented in recent LightGBM-based pipelines [28].

## 3. Results

### 3.1. Exploratory Analysis: The Closed-Loop Compensation Phenomenon

**Figure 4:**
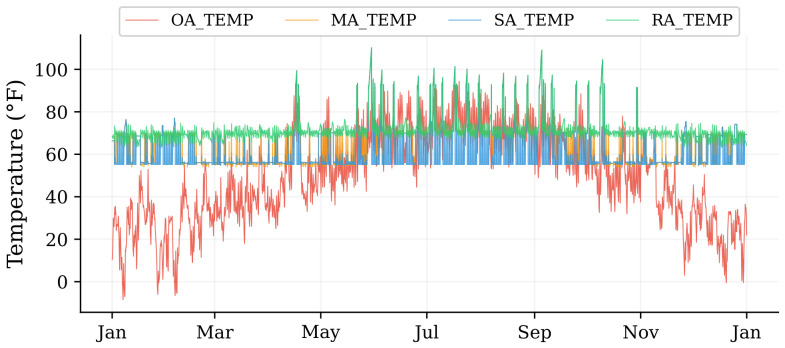
Annual fault-free temperature traces for the SD-AHU system. Supply air temperature (SA_TEMP) remains tightly regulated around its setpoint throughout the year, while mixed air (MA_TEMP) and outdoor air (OA_TEMP) temperatures follow seasonal patterns. This controlled variation provides rich inter-sensor correlation structure exploitable by virtual-sensor models.

Figure 4 presents the annual fault-free temperature time series for OA, MA, SA, and RA, demonstrating consistent seasonal patterns and tightly regulated supply air temperature near its setpoint. These stable baselines provide a foundation for the temporal cross-correlations utilized by the virtual-sensor models.

**Figure 5:**
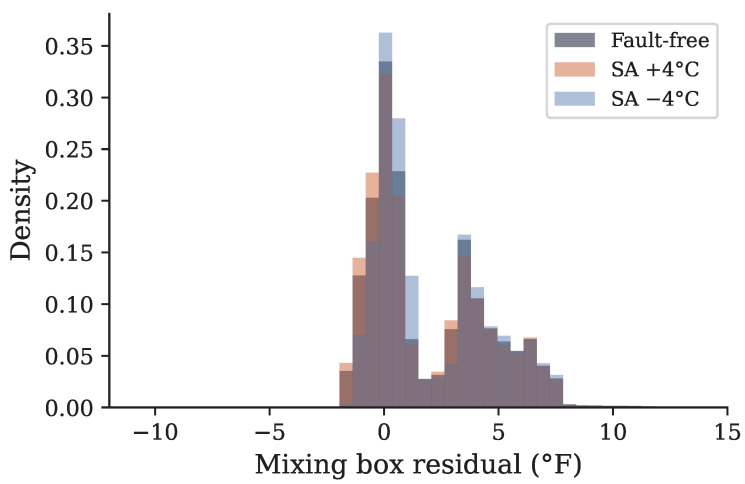
Mixing box residual distributions for the fault-free baseline and two representative SA_TEMP sensor bias scenarios (SA +4 °C and SA −4 °C). The near-complete overlap demonstrates that physics-based energy balance residuals cannot detect sensor bias when closed-loop control compensates for the bias in the reading. The shift-to-noise ratio remains below 0.06 for all four bias scenarios.

Figure 5 displays the mixing box residual distributions for the fault-free baseline and two representative SA_TEMP sensor bias scenarios (SA +4 °C and SA −4 °C). The distributions nearly coincide—the shift-to-noise ratio, defined as the difference in mean residual between the fault and fault-free scenarios divided by the fault-free residual standard deviation, stays below 0.06 at every bias level—confirming that physics-based residuals are blind to sensor bias under closed-loop control. The controller restores both the mixed air temperature and its residual to near-fault-free values simply by adjusting the cooling coil valve.

**Figure 6:**
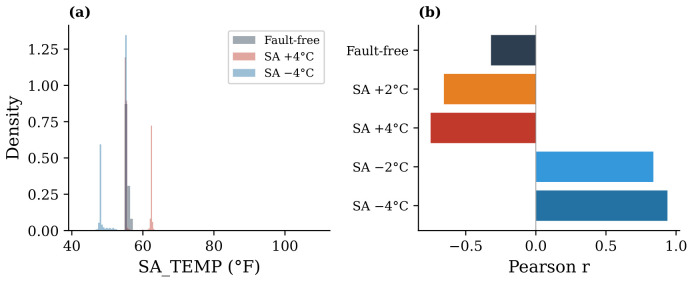
Closed-loop compensation effect. (**a**) SA_TEMP distributions for the fault-free, SA +4 °C, and SA −4 °C scenarios are nearly indistinguishable, illustrating how the controller conceals the bias by adjusting actuators even at the highest bias magnitudes. (**b**) The SA_TEMP–MA_TEMP Pearson correlation shifts dramatically across all bias scenarios (ranging from r=−0.74 for SA +2 °C to r=+0.96 for SA −4 °C), revealing the fault through disrupted inter-sensor consistency despite unchanged marginal sensor distributions.

Figure 6 makes the compensation mechanism visible. Panel (a) shows that the SA_TEMP distributions remain within the same operational range across the fault-free, SA +4 °C, and SA −4 °C scenarios; although minor differences are visible upon close inspection, a conventional threshold monitor examining SA_TEMP alone would not reliably distinguish fault from fault-free conditions. The controller substantially conceals the bias by adjusting actuators. Panel (b) tells a different story: the Pearson correlation between SA_TEMP and MA_TEMP swings from r=−0.33 (fault-free) to r=−0.74 (SA +2 °C) and r=+0.86 (SA −2 °C), and reaches r=+0.96 for the strongest bias (SA −4 °C). The marginal distribution looks normal; the inter-sensor coherence does not.

**Figure 7:**
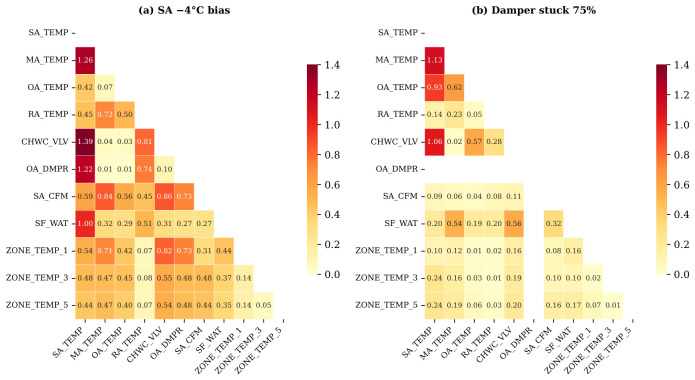
Multi-pair correlation disruption heatmaps; each cell shows $|r_{\mathrm{fault}}-r_{\mathrm{fault}\text{-}\mathrm{free}}|$, the absolute change in pairwise Pearson correlation relative to the fault-free baseline. (**a**) SA −4 °C sensor bias disrupts 20 sensor pairs, with the largest deviations concentrated around SA_TEMP and its thermodynamic neighbors. (**b**) Damper-stuck 75% produces a qualitatively different disruption pattern centered on MA_TEMP and outdoor-air-related channels. These distinct fingerprints motivate the per-sensor anomaly ranking used for fault isolation.

Figure 7 compares the cross-sensor disruption patterns for SA −4 °C sensor bias and Damper stuck at 75%. The sensor bias disrupts 20 sensor pairs, concentrated around SA_TEMP and its thermodynamic neighbors (MA_TEMP, ZONE_TEMP_1). The mechanical fault disrupts a different subset, centered on MA_TEMP and OA-related channels—an orthogonal disruption fingerprint.

**Figure 8:**
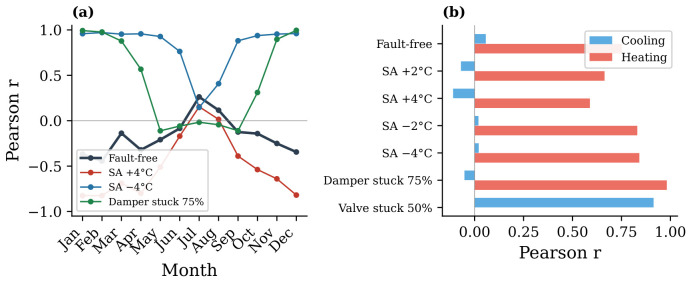
Temporal instability of single-pair correlations. (**a**) Monthly SA_TEMP–MA_TEMP Pearson correlation for all scenarios, showing a near-zero dead zone during summer months (July–August) in the fault-free baseline. (**b**) Correlation strength by HVAC operating mode (heating versus mechanical cooling) confirms that no single sensor pair provides uniformly informative anomaly signals across all operating conditions.

Figure 8 tracks the SA_TEMP–MA_TEMP pairwise correlation across months and operating modes. During summer, the correlation drops to near zero—the supply air setpoint makes the mixing process thermodynamically inactive. This dead zone, visible in both the monthly trajectories (panel a) and the by-mode breakdown (panel b), shows that relying on any single sensor pair would yield unreliable detection during parts of the year. The multi-pair virtual sensor approach addresses this limitation by leveraging correlations across diverse sensor neighborhoods, with only a subset entering the dead zone at any given time. Monthly SA_TEMP–MA_TEMP Pearson correlations for representative scenarios are provided in Table 5.

### 3.2. Detection Performance

Each fault scenario spans the same full-year period as the fault-free baseline. Evaluation uses only the validation period (the last 20% of occupied timesteps and the corresponding calendar dates). For each scenario, the negative class consists of the fault-free validation days, and the positive class consists of the same date range from the fault scenario; the two classes therefore contain the same number of days. The AUC is computed from the receiver operating characteristic (ROC) curve generated by varying the detection threshold over all observed daily system-level z-score values using sklearn.metrics.roc_curve and auc. Precision and recall are reported at the fixed z=3 operating threshold. A day is classified as positive (faulty) if $Z_{d}>3$; a true positive is a fault-scenario day correctly classified as positive, and a false positive is a fault-free day incorrectly classified as positive.

**Figure 9:**
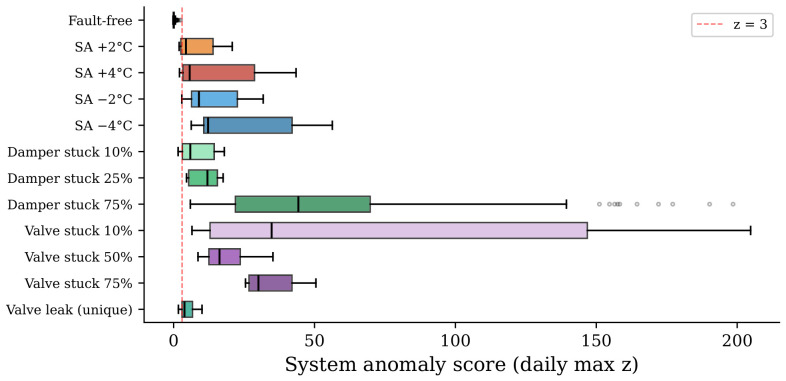
Distribution of daily system anomaly scores across all 12 SD-AHU scenarios. Each box shows the interquartile range of maximum daily z-scores; whiskers extend to the most extreme data point within 1.5× the interquartile range, and points beyond this range are plotted as individual outliers. The fault-free baseline clusters tightly near zero, while all sensor bias and mechanical-fault scenarios produce substantially elevated scores, confirming strong separation between normal and faulted conditions.

Figure 9 presents box plots of daily system anomaly scores for all 12 SD-AHU scenarios. The fault-free distribution is tightly concentrated near z=0. In contrast, all sensor bias and mechanical fault scenarios yield distributions that are substantially elevated and clearly separated from the fault-free baseline.

**Figure 10:**
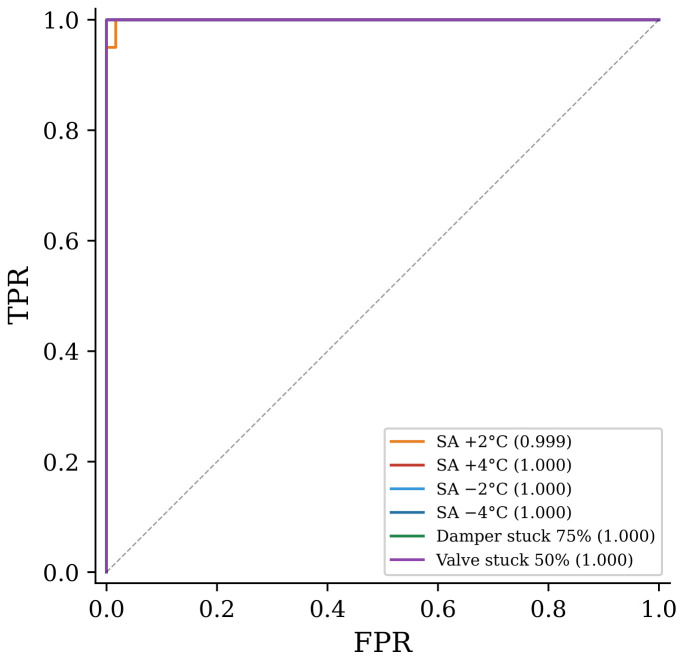
ROC curves for selected SD-AHU fault scenarios. The proposed virtual-sensor method achieves near-perfect discrimination for all scenarios. The mildest sensor bias (SA +2 °C, AUC = 0.9992) represents the lower bound of performance, with all other scenarios at AUC = 1.0.

Table 6 reports AUC, precision, and recall at z=3 for every evaluated SD-AHU scenario. The mildest bias (SA +2 °C), yields precision 1.0 and recall 0.833. SA +4 °C achieves AUC = 1.0 with recall 0.983; all remaining scenarios achieve perfect precision and recall. The ROC curves in Figure 10 confirm this.

No fault-free validation day was misclassified under any scenario (false-positive rate = 0.0).

**Figure 11:**
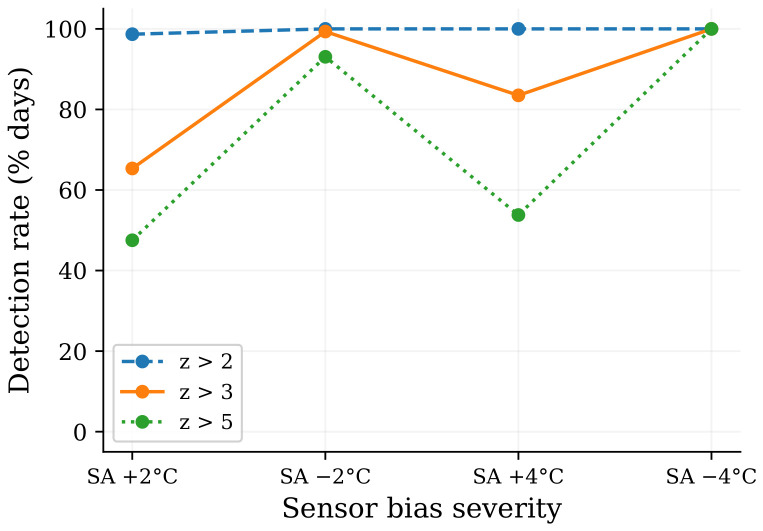
Detection rate as a function of SA_TEMP bias severity at three anomaly thresholds (z=2, 3, 5). The detection rate increases monotonically with the magnitude of the bias. At the z=3 operating threshold, recall reaches 83.3% for the mildest (+2 °C) scenario and 98.3% for the +4 °C scenario; all ±4 °C biases are detected with 100% recall.

The five scenarios omitted from Table 6 (Damper stuck 10%, 25%; Valve stuck 10%, 75%; Valve leak) all achieve AUC = 1.0 with perfect precision and recall. Figure 11 plots detection rate against bias severity at three thresholds (z=2,3,5). Detection climbs monotonically with bias magnitude. The ±4 °C scenarios are detected with 100% recall even at the most conservative threshold, while the +2 °C scenario achieves 83.3% recall at z=3.

### 3.3. Fault Isolation Results

Table 7 lists, for each of the 12 SD-AHU scenarios, the top-ranked sensor, its median daily anomaly score, and the isolation margin. For the larger biases (±4 °C), SA_TEMP ranks first with margins of 0.38 (SA +4 °C) and 0.87 (SA −4 °C). For the milder biases (±2 °C), MA_TEMP ranks first with SA_TEMP at rank 2; the margins are 0.91 (SA +2 °C) and 0.57 (SA −2 °C). This ranking instability at ±2 °C is expected: the closed-loop compensation nearly masks the bias, making the competition between SA_TEMP and its nearest thermal neighbor (MA_TEMP) sensitive to model variance. A robustness analysis across 10 random seeds (Section 4.3) quantifies this sensitivity. For Damper stuck 75%, MA_TEMP tops the ranking (score = 44.27, margin = 28.57)—physically expected, since a stuck outdoor air damper disrupts mixing box temperature before any downstream effect reaches supply air. Valve-stuck scenarios place SA_TEMP first, consistent with the direct causal path from valve to supply air temperature.

**Figure 12:**
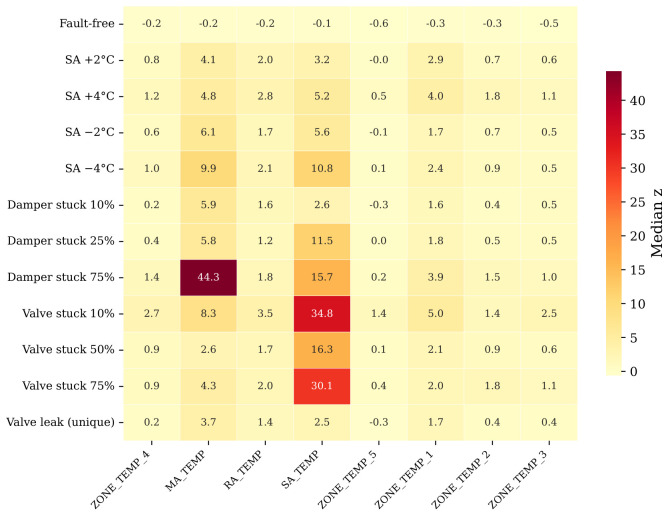
Per-sensor median anomaly score heatmap for all SD-AHU scenarios. Rows correspond to fault scenarios; columns to temperature sensors. Darker cells indicate higher anomaly concentration. The heatmap confirms physically interpretable isolation: sensor bias scenarios consistently highlight SA_TEMP, while the large damper fault (Damper stuck 75%) shifts the peak to MA_TEMP.

Figure 12 displays the isolation results as a heatmap of per-sensor median anomaly scores across all scenarios. The observed visual structure supports the physical interpretability of the rankings. Specifically, sensor bias scenarios concentrate anomaly energy on SA_TEMP and its immediate thermal neighbors, whereas mechanical-fault scenarios shift this energy toward MA_TEMP in the case of damper faults or toward the valve-side channels.

### 3.4. Fault Type Discrimination Results

Table 8 presents the three-dimensional coordinates for each scenario within the discrimination space, defined by system anomaly score, Damper_MAE, and Valve_MAE. The four sensor bias scenarios demonstrate Damper_MAE values approximately equal to zero and Valve_MAE values less than or equal to 0.0017, which suggests that the actuators maintain normal tracking error. The three damper fault scenarios exhibit Damper_MAE values ranging from 0.346 to 0.581, with Valve_MAE values approximately zero. The four valve fault scenarios display Valve_MAE values between 0.033 and 0.750, with Damper_MAE equal to zero.

**Figure 13:**
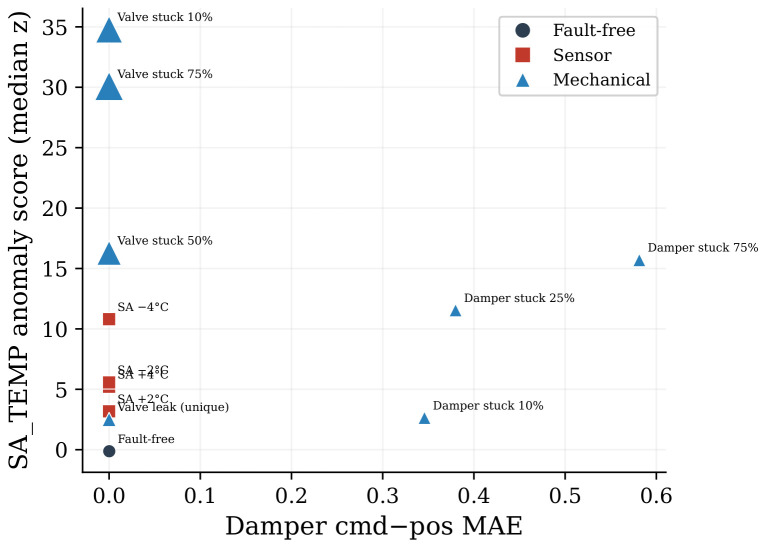
Fault type discrimination scatter plot (SA_TEMP anomaly score vs. Damper cmd-pos MAE, colored by fault category). The fault-free baseline (dark slate) sits near the origin. Sensor faults (red) cluster at near-zero Damper MAE with moderate anomaly scores. Mechanical faults (blue) form two distinct subclusters: damper faults exhibit elevated Damper MAE, while valve faults exhibit high anomaly scores with near-zero Damper MAE.

Figure 13 illustrates the three-cluster structure within the discrimination space. The three fault categories occupy distinct, non-overlapping regions separated by zero-width margins along both actuator MAE dimensions. This result confirms that command-position discrepancy analysis achieves perfect fault type discrimination for the SD-AHU scenarios evaluated.

### 3.5. Baseline Comparison

Table 9 compares detection AUC across the three methods. The mixing box residual yields AUC = 0.33–0.53 in sensor bias scenarios, essentially no better than chance, confirming its failure under closed-loop compensation. PCA achieves an AUC of 1.0 across all SD-AHU scenarios tested. The proposed method matches PCA in 10 of 11 scenarios and falls short by only 0.0008 on SA +2 °C (AUC = 0.9992 vs. 1.0).

**Figure 14:**
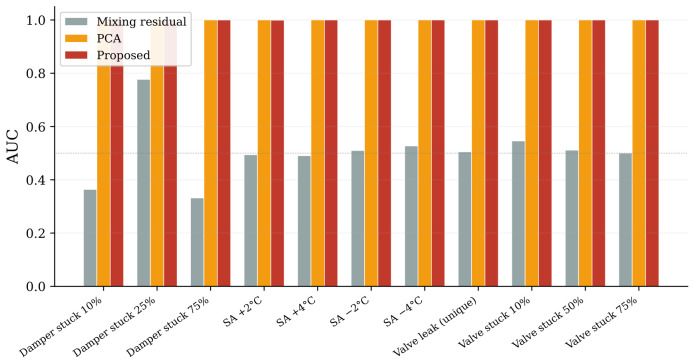
Detection AUC comparison for SD-AHU scenarios: mixing box residual (gray), PCA (orange), and proposed virtual-sensor method (red). The mixing box residual fails for all sensor bias scenarios. PCA and the proposed method achieve equivalent detection AUC on SD-AHU, but the proposed method also provides fault isolation and discrimination capabilities.

Figure 14 visualizes these numbers. PCA matches the proposed method in SD-AHU detection, but it cannot isolate individual sensors or discriminate fault types—its output is a single scalar reconstruction error aggregated across all sensors.

### 3.6. Cross-System Validation: Fan Coil Unit

Table 10 compares the detection AUC on the FCU system. The proposed method achieves AUC = 1.0 across all four room-temperature bias scenarios; PCA achieves only 0.634–0.897 in the same cases. On Cooling valve stuck 50% and OA damper stuck 100%, both methods approach AUC = 1.0.

Table 11 gives the FCU isolation rankings. FCU_RAT (return air temperature, measuring the same air volume as the biased room sensor) ranks first in all four sensor bias cases, with scores from 52.51 to 106.27. Mechanical fault isolation follows physical causality: FCU_CLG_RWT for cooling valve faults, FCU_HTG_RWT for heating valve faults, FCU_MAT for damper faults.

We note that these results reflect subsystem-level, not sensor-level, isolation. FCU_RAT and RM_TEMP both measure the conditioned zone air; identifying FCU_RAT correctly localizes the fault to the zone temperature subsystem. Disambiguating co-located sensors within a single thermal zone would require additional logic, e.g., analyzing residual sign or comparing values against the zone setpoint. Subsystem isolation is reliable; sensor-level disambiguation within a subsystem remains open.

**Figure 15:**
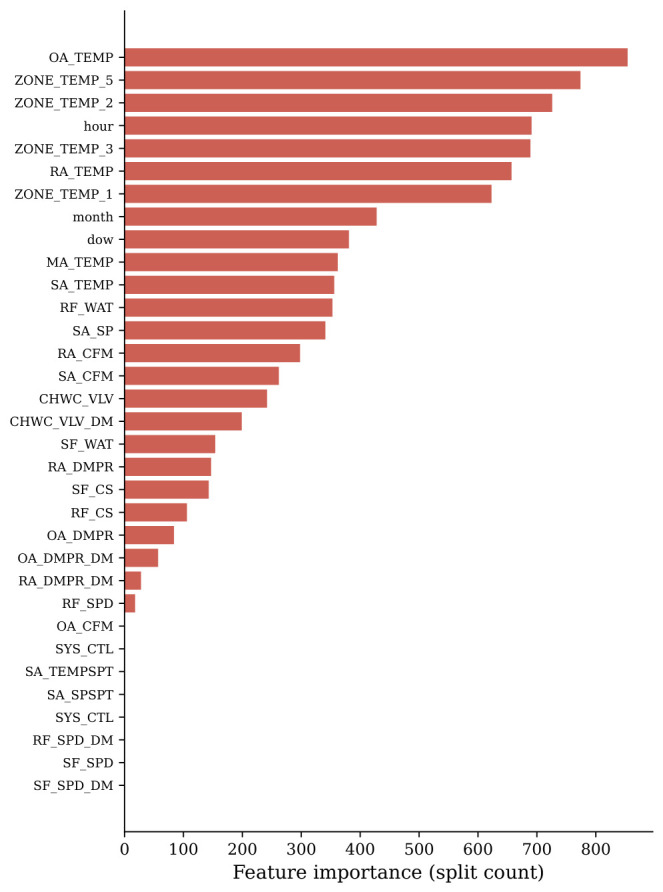
LightGBM feature importance for the SA_TEMP virtual-sensor model (split count). Mixed air temperature (MA_TEMP) and return air damper position (RA_DMPR) are the dominant predictors, followed by cooling coil valve position (CHWC_VLV) and its command signal (CHWC_VLV_DM). The importance distribution confirms that the BRICK-derived feature neighborhood captures physically meaningful thermodynamic relationships. SYS_CTL appears at two bar positions because it is present both as an explicit occupancy context feature and as a passthrough column from the BRICK-derived column set; the total split count is additive.

Figure 15 ranks the LightGBM features for the SA_TEMP model by split count. MA_TEMP and RA_DMPR dominate, followed by CHWC_VLV and its command signal CHWC_VLV_DM. This ordering mirrors the AHU thermodynamics: supply air temperature depends primarily on the mixing process (MA_TEMP, damper) and the cooling coil (valve position). Zone temperatures contribute moderately, coupled to supply air only indirectly through the return stream. Temporal features (hour, month, day of week) appear mid-range, confirming that diurnal and seasonal patterns carry predictive value. The distribution of feature importance indicates that the BRICK-derived feature neighborhood captures physically meaningful relationships rather than spurious correlations.

**Figure 16:**
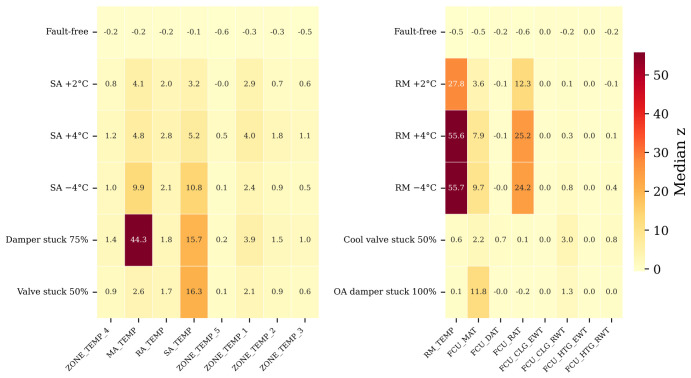
Cross-system isolation heatmaps: (**left**) SD-AHU, (**right**) FCU. Both systems were processed by the same pipeline function with no system-specific modifications. The spatial structure of anomaly concentration follows the physical topology of each system, confirming that BRICK-automated feature derivation produces thermodynamically coherent virtual-sensor models across different HVAC configurations.

Figure 16 displays side-by-side isolation heatmaps for the SD-AHU and FCU systems, demonstrating that the BRICK-automated pipeline generates comparable isolation structures across two physically distinct HVAC topologies.

## 4. Discussion

### 4.1. The Closed-Loop Compensation Phenomenon as a Detection Barrier

The exploratory analysis demonstrates that the closed-loop compensation mechanism represents a fundamental property of well-regulated HVAC systems, rather than an exceptional case. Threshold checks, statistical process control limits, physics-based residuals, and basically any method that examines the biased sensor in isolation will be circumvented whenever the control loop operates as designed. The observed mixing box residual AUC of 0.33 to 0.53 for SA_TEMP sensor bias scenarios provides quantitative evidence of this limitation.

The virtual-sensor approach addresses this limitation by indirectly evaluating the biased sensor through the consistency of the broader inter-sensor network. This mechanism accounts for the systematic failure of single-sensor threshold monitoring, even when sensors are physically biased. Because the control loop restores each sensor’s marginal distribution to normal, the fault signature exists only in the joint distribution of correlated sensors—precisely where the per-sensor virtual model looks. This same principle explains why PCA fails on the FCU bias scenarios: its global reconstruction error averages disruptions across all sensor pairs, diluting a fault that affects only a local subset of relationships. In contrast, the per-sensor virtual model attributes residuals directly to the sensor with degraded predictions, providing the diagnostic resolution identified as necessary for closed-loop systems by Kim and Katipamula [2] and operationalized by Chen et al. through cross-level fault diagnosis, where a primary subsystem sensor error propagates into anomalous behavior in downstream AHU components [29].

### 4.2. Novelty and Positioning Against the Literature

The primary contribution of this study is the automation of virtual-sensor feature selection using BRICK metadata. Previous virtual-sensor approaches have demonstrated strong per-sensor fault isolation, as seen in Gao et al.’s LSTM-based chiller sensors [18], Liu et al.’s Bayesian inference framework [19], and Elnour et al.’s auto-associative reconstruction approach [22]. However, each of these methods requires an expert to manually determine which sensors to use for prediction. The BRICK graph traversal developed in this work replaces expert-driven selection with a systematic metadata query, thereby extending portability across multiple buildings without the need for re-engineering.

This framework is distinguished from three categories of related work. Metadata-driven diagnostic methods, such as Gao et al. (2024) using Project Haystack to auto-construct a Bayesian network [24], Hwang et al.’s FSBrick extension of BRICK for fault-symptom encoding [15], and Hosamo et al.’s BRICK-based digital twin [26], utilize metadata to structure building models but do not define data-driven prediction neighborhoods. Hosamo et al., for instance, employ BRICK for data integration, but the FDD logic itself is hand-coded APAR rules [26]. Gao et al. (2024) explicitly exclude sensor faults and flag sensor bias as future work [24]. Virtual-sensor FDD methods without metadata automation, including those of Gao et al. [18], Liu et al. [19], Elnour et al. [22], and the FADED framework [25], achieve strong per-sensor isolation but demand either manual configuration or physical-sensor deployment. FADED, for instance, classifies VAV sensor faults with 99.57% accuracy [25] but only after a technician walks the building for 30 min with a wireless anemometer and Bluetooth sensor. No virtual model is built; no metadata standard is applied. Our framework eliminates both manual neighborhood specification and the need for physical sensing hardware. Ren et al.’s thermodynamic-law-integrated autoencoder [30] claims multi-system applicability but was validated on a single system type only. In contrast, the present deployment on SD-AHU and FCU, using the same pipeline function without system-specific modifications, provides empirical evidence of broader applicability.

Several additional recent methods further contextualize the proposed framework. In the chiller domain, Fan et al. demonstrated that SVM-based fault diagnosis can achieve 97.68% accuracy using only factory-installed sensors on the ASHRAE RP-1043 benchmark [31], and subsequently showed that PCA-SMOTE oversampling enables cross-system transfer of diagnostic knowledge from a centrifugal chiller to a screw chiller with 96.70% accuracy [32]. While these methods address data scarcity for mechanical faults, they do not target sensor faults and require labeled training data. For sensor calibration, Yoon [20] and Choi and Yoon [21] demonstrated that coupling virtual sensors with Bayesian inference can correct in situ sensor bias with high accuracy, but both were validated only on simulated single-system data and require manual selection of virtual sensor inputs. Papadopoulos et al.’s distributed architecture [16] addresses scalability through a modular agent network but requires explicit physical models for each zone. The proposed framework is unique in automating the virtual sensor construction step through BRICK graph traversal, enabling deployment across heterogeneous systems without manual configuration, labeled fault data, or physical modeling.

Table 12 summarizes the distinguishing characteristics of the most closely related approaches.

We compare against PCA and a physics-based mixing box residual, but omit deep learning methods (autoencoders, LSTM anomaly detectors). This is deliberate: the contribution is the metadata-automated framework, not the regression algorithm. LightGBM is the prediction engine, but the framework is model-agnostic—any regressor that can learn inter-sensor relationships from fault-free data could replace it without affecting target selection, z-score scoring, or isolation ranking. LightGBM was chosen due to its rapid training, competitive accuracy without extensive hyperparameter tuning, and interpretable feature importance output.

Bi et al.’s review of 211 FDD studies (2013–2023) ranks LightGBM as the top-performing ensemble method for HVAC FDD, above Random Forest, XGBoost, and SVM, with a macro-average F1 of 0.88 [33]. In this study, LightGBM is employed as the regression backbone for virtual sensor construction rather than as a direct fault classifier, representing a complementary application within the same algorithm family. This approach leverages LightGBM’s capacity to capture nonlinear inter-sensor relationships in the fault-free regime, rather than its classification capabilities for labeled fault patterns. The semi-supervised framework, which involves training exclusively on fault-free data, directly addresses the data scarcity challenge identified as a primary obstacle to AI-based FDD deployment [9,34].

### 4.3. Limitations

The current evaluation is subject to several limitations. The analysis relies exclusively on simulated data from EnergyPlus-Modelica and HVACSIM+. In contrast, real-building data would introduce measurement noise, missing values, sensor drift, and occupancy-driven dynamics that are not fully captured by physics-based simulation. Second, only temperature bias faults were tested; pressure sensor bias, flow meter faults, and gradual drift remain unexamined. At the mildest biases (±2 °C), closed-loop compensation suppresses the SA_TEMP anomaly enough that its nearest thermal neighbor MA_TEMP can overtake it in the isolation ranking. In real buildings, where controllers are tuned differently, this competition between the biased sensor and its thermal neighbors may be even more pronounced.

At the reference seed (random_state = 42), SA_TEMP ranks first for the ±4 °C biases but MA_TEMP ranks first for the ±2 °C biases, with isolation margins of 0.91 and 0.57, respectively. To quantify the sensitivity of these rankings to stochastic model variance, we retrained all models with 10 different random seeds (0–9). Detection AUC remained stable: the median AUC for SA +2 °C was 0.9999 (range 0.9975–1.0), and all other scenarios achieved AUC = 1.0 at every seed. Recall at z=3 for SA +2 °C had a median of 0.958 (range 0.783–1.0). Isolation was more variable: the SA +2 °C margin ranged from 0.29 to 2.27 (median 0.99), and the SA −2 °C margin ranged from 0.01 to 1.54 (median 0.79). For the ±4 °C cases, margins were wider: SA +4 °C median 2.50 (range 1.74–3.50) and SA −4 °C median 1.94 (range 0.09–6.09). These results confirm that detection performance is robust to model variance, while isolation ranking at ±2 °C is sensitive to the random subsampling in LightGBM. The minimum bias severity at which isolation remains reliable has not been characterized and represents an avenue for future research.

The FCU BRICK model employs class names that do not directly correspond to CSV column headers, necessitating the fallback heuristic described in Section 2.3. This mismatch reflects an inconsistency in the published benchmark rather than a limitation of the BRICK standard itself, underscoring the importance of consistent naming conventions in BRICK model development. Additionally, the 80/20 temporal split assigns the validation period to the second half of the year (primarily winter and early spring), leaving summer performance partially uncharacterized for systems exhibiting strong seasonal variation.

Only SA_TEMP bias scenarios exist in the SD-AHU data; the OA bias files duplicate the fault-free baseline, precluding OA fault evaluation. Expanding the evaluation to a broader set of sensor locations would enhance the generalizability of the findings.

The framework assumes the availability of a fault-free training period. In practice, this can be obtained from a commissioning window, a period of known-good operation verified by inspection, or a maintenance-confirmed baseline. If the training data is contaminated by undetected faults, the models will learn the faulty regime as normal and detection sensitivity will degrade. The sensitivity of LightGBM predictions to training data contamination has not been quantified in this study and represents an avenue for future work.

The exclusive reliance on simulated data (EnergyPlus-Modelica for the SD-AHU, HVACSIM+ for the FCU) constitutes a further limitation. Real-building data would introduce measurement noise, communication dropouts, missing values, sensor drift, and occupancy-driven variability that are not fully captured by physics-based simulation. Validating the framework on operational-building data with naturally occurring faults is the immediate priority for future work.

### 4.4. Practical Deployment Considerations

The implementation of the proposed framework in actual building management systems presents several practical challenges. Sensor measurements in operational settings are often affected by noise, communication delays, and occasional data loss, which necessitates robust preprocessing and data validation strategies. Furthermore, BRICK metadata models in real-world buildings may be incomplete or inconsistently defined, highlighting the need for automated validation or enrichment methods. From a computational standpoint, the low inference cost of the proposed approach enables real-time deployment; however, periodic model retraining may be necessary to address system aging and seasonal variations. Integration with existing building management system (BMS) platforms also requires careful consideration of data access, interoperability, and system constraints. From a scalability perspective, training all eight SD-AHU virtual-sensor models on approximately 220,000 occupied timesteps required under two minutes on a single CPU core, and scoring one day of data across all models took under one second. Both operations scale linearly with the number of systems and are trivially parallelizable. For a portfolio of *N* systems, the total training time is approximately 2N minutes on a single core, or under two minutes on *N* cores. The primary bottleneck at scale is therefore not computational cost but the availability and quality of BRICK metadata models across the building portfolio.

### 4.5. Directions for Further Investigation

An avenue for further research would be to validate the framework on real building data that exhibit naturally occurring sensor drift and noise, with a focus on LightGBM residual behavior during the gradual onset of bias. Expanding anomaly scoring to include pressure and flow sensor channels is a logical progression, as these channels are commonly biased in actual AHU systems and are included in the LBNL benchmark. The seven remaining LBNL system types (dual-duct AHUs, packaged rooftop units, fan-powered units, boiler plants, chiller plants) offer a ready-made evaluation platform for testing cross-system generalization with the same pipeline function. Implementing adaptive model updating to monitor seasonal drift in fault-free sensor relationships would address the previously identified temporal limitations.

## 5. Conclusions

We have presented a framework for automated temperature sensor fault detection, isolation, and discrimination in smart-building HVAC systems—one that requires neither labeled fault data nor manual sensor neighborhood selection. BRICK schema metadata served as the configuration layer: traversing the .ttl graph determines, for each temperature sensor, which correlated sensors feed the LightGBM virtual model trained on fault-free data. Z-scored prediction residuals drive detection; per-sensor anomaly ranking drives isolation; actuator command-position discrepancy drives fault type discrimination.

On the LBNL benchmark, AUC reaches 0.9992 for the mildest bias (SA +2 °C) and 1.0 for every other SD-AHU scenario; the biased sensor (SA_TEMP) ranks first for the ±4 °C biases and second for ±2 °C, where its nearest thermal neighbor (MA_TEMP) ranks first; detection AUC remains above 0.997 across 10 random seeds, confirming robustness to model variance. On the FCU, the same pipeline, with no modification, achieves AUC = 1.0 on all room-temperature bias scenarios, where PCA reaches only 0.63–0.90. Sensor, damper, and valve faults separate into non-overlapping clusters in the command-position discrepancy space.

Three practical findings stand out. First, single-point energy balance residuals, such as the mixing box equation evaluated in this study, fail to detect supply air temperature sensor bias because the biased sensor does not appear as a variable in the energy balance, and the controller’s compensatory actuator adjustments shift both sides of the equation approximately together, leaving the residual non-discriminative; monitoring inter-sensor consistency through virtual-sensor residuals addresses this limitation, as the fault signature remains visible in the joint distribution of correlated sensors even when it is invisible in any single sensor’s marginal distribution. Second, per-sensor anomaly ranking localizes faults in a physically interpretable way: in every scenario, the top-ranked sensor matches the expected fault location. Third, BRICK metadata enabled deployment of the same pipeline function (derive_targets()) across two physically distinct HVAC systems (a single-duct AHU and a four-pipe FCU) without system-specific code changes; the column-name fallback activated automatically for the FCU where BRICK entity names did not match CSV headers (Section 2.3). Taken together, these results confirm that BRICK-automated virtual sensing affords a scalable path toward sensor-fault-aware building diagnostics.

## 6. Reproducibility

The experiments in this study were conducted using the publicly available LBNL FDD benchmark dataset. The proposed framework was implemented using standard Python 3.12 libraries, including LightGBM for model training and RDF processing tools for BRICK metadata parsing. All preprocessing steps, model configurations, and evaluation procedures are described in Section 2 to ensure reproducibility. 

## Figures and Tables

**Table 1:** Abbreviations, mathematical notation, and metrics used in the paper.

**General Abbreviations**			
HVAC	Heating, Ventilation, and Air Conditioning	FDD	Fault Detection and Diagnosis
AHU	Air Handling Unit	FCU	Fan Coil Unit
BMS	Building Management System	BRICK	Building Metadata Schema
LBNL	Lawrence Berkeley National Laboratory	PCA	Principal Component Analysis
AUC	Area Under the ROC Curve	ROC	Receiver Operating Characteristic
TPR	True-Positive Rate	FPR	False-Positive Rate
MAE	Mean Absolute Error	RMSE	Root Mean Squared Error
PID	Proportional–Integral–Derivative controller	TTL	RDF Turtle File Format
**HVAC Variable Abbreviations**			
SA_TEMP	Supply Air Temperature	MA_TEMP	Mixed Air Temperature
RA_TEMP	Return Air Temperature	OA_TEMP	Outdoor Air Temperature
ZONE_TEMP_i	Zone Temperature (zone *i*)	OA_DMPR	Outdoor Air Damper Position
OA_DMPR_DM	Outdoor Air Damper Command	RA_DMPR	Return Air Damper Position
RA_DMPR_DM	Return Air Damper Command	CHWC_VLV	Cooling Coil Valve Position
CHWC_VLV_DM	Cooling Coil Valve Command	SA_CFM	Supply Air Flow
OA_CFM	Outdoor Air Flow	RA_CFM	Return Air Flow
SF_SPD	Supply Fan Speed	RF_SPD	Return Fan Speed
SF_WAT	Supply Fan Power	RF_WAT	Return Fan Power
SYS_CTL	System Operation Indicator		
**Mathematical Notation and Metrics**			
**Symbol**	**Description**		
$y_{t,s}$	Actual value of sensor *s* at time *t*		
${\hat{y}}_{t,s}$	Predicted value of sensor *s* at time *t*		
$e_{t,s}$	Absolute residual $|y_{t,s}-{\hat{y}}_{t,s}|$		
$\mu_{s}$	Mean residual under normal conditions		
$\sigma_{s}$	Standard deviation of residuals		
$z_{t,s}$	Z-scored residual		
$Z_{d,s}$	Daily anomaly score for sensor *s*		
$Z_{d}$	System-level anomaly score ($\max_{s}Z_{d,s}$)		
${\tilde{Z}}_{s}$	Median anomaly score over a scenario		
F(s)	Feature set associated with sensor *s*		
α	Outdoor air damper fraction		
*t*	Time index		
*s*	Sensor index		
*d*	Day index		
Damper_MAE	Damper command-position error		
Valve_MAE	Valve command-position error		

**Table 2:** SD-AHU scenario inventory. All scenarios share 525,540 rows and 30 columns at 1-min resolution.

Scenario	Fault Type	Rows
Fault-free	Fault-free	525,540
SA +2 °C	Sensor	525,540
SA +4 °C	Sensor	525,540
SA −2 °C	Sensor	525,540
SA −4 °C	Sensor	525,540
Damper stuck 10%	Mechanical	525,540
Damper stuck 25%	Mechanical	525,540
Damper stuck 75%	Mechanical	525,540
Valve stuck 10%	Mechanical	525,540
Valve stuck 50%	Mechanical	525,540
Valve stuck 75%	Mechanical	525,540
Valve leak	Mechanical	525,540

**Table 3:** BRICK entity inventory for the SD-AHU system: A total of 41 entities, comprising equipment, zones, sensors, commands, and setpoints.

Entity	BRICK Class	Outgoing Edges ^2^
AHU	AHU	21
Cooling_Coil	Chilled_Water_Coil	2
Outdoor_Air_Damper	Outside_Damper	2
Return_Air_Damper	Return_Damper	2
Supply_Air_Fan	Fan	4
Return_Air_Fan	Fan	4
Zone_1 – Zone_5	HVAC_Zone	1 each
SA_TEMP	Supply_Air_Temperature_Sensor	0
MA_TEMP	Mixed_Air_Temperature_Sensor	0
RA_TEMP	Return_Air_Temperature_Sensor	0
OA_TEMP	Outside_Air_Temperature_Sensor	0
ZONE_TEMP_1–5	Zone_Air_Temperature_Sensor	0 each
OA_DMPR	Damper_Position_Sensor	0
OA_DMPR_DM	Damper_Position_Command	0
RA_DMPR	Damper_Position_Sensor	0
RA_DMPR_DM	Damper_Position_Command	0
CHWC_VLV	Valve_Position_Sensor	0
CHWC_VLV_DM	Valve_Position_Command	0
SA_TEMPSPT	Supply_Air_Temperature_Setpoint	0
SA_CFM, OA_CFM, RA_CFM	Flow sensors	0
SF_SPD, RF_SPD	Speed status	0
SF_WAT, RF_WAT	Electrical_Power_Sensor	0

^2^ Number of hasPoint, hasPart, and feeds edges originating from this entity. Sensors show 0 because they are leaf nodes: equipment points to sensors via hasPoint, but sensors do not point to other entities.

**Table 4:** Virtual-sensor training performance on the fault-free validation set (SD-AHU).

Target Sensor	RMSE (°F)	MAE (°F)	AbsRes Std (°F)
SA_TEMP	0.1516	0.0388	0.1465
MA_TEMP	0.1919	0.0910	0.1689
RA_TEMP	0.1266	0.0714	0.1045
ZONE_TEMP_1	0.2238	0.1355	0.1781
ZONE_TEMP_2	0.5980	0.3886	0.4545
ZONE_TEMP_3	0.5474	0.3955	0.3785
ZONE_TEMP_4	0.4995	0.3310	0.3741
ZONE_TEMP_5	0.9865	0.7213	0.6730

**Table 5:** Monthly SA_TEMP–MA_TEMP Pearson correlation for the fault-free baseline and six representative fault scenarios. Positive correlations indicate disrupted inter-sensor relationships; negative values are normal (supply air is cooled below mixed air). In the summer months (July = 7, August = 8), the fault-free case exhibits near-zero correlation, reflecting the thermodynamic dead zone during economizer-dominated operation.

Scenario	Jan	Feb	Mar	Apr	May	Jun	Jul	Aug	Sep	Oct	Nov	Dec
Fault-free	−0.37	−0.44	−0.14	−0.32	−0.21	−0.08	+0.26	+0.12	−0.12	−0.14	−0.25	−0.34
SA +2 °C	−0.74	−0.76	−0.53	−0.69	−0.39	−0.15	+0.06	−0.03	−0.30	−0.41	−0.55	−0.72
SA +4 °C	−0.83	−0.82	−0.69	−0.80	−0.51	−0.17	+0.15	+0.02	−0.39	−0.54	−0.64	−0.82
SA −2 °C	+0.86	+0.91	+0.85	+0.86	+0.74	+0.46	+0.04	+0.12	+0.60	+0.78	+0.82	+0.86
SA −4 °C	+0.96	+0.97	+0.95	+0.96	+0.93	+0.76	+0.15	+0.41	+0.88	+0.94	+0.95	+0.96
Valve stuck 50%	+0.92	+0.94	+0.86	+0.92	+0.86	+0.75	+0.06	+0.48	+0.84	+0.89	+0.92	+0.92
Damper stuck 75%	+0.99	+0.98	+0.88	+0.57	−0.11	−0.06	−0.02	−0.04	−0.11	+0.31	+0.90	+1.00

**Table 6:** Detection metrics for SD-AHU fault scenarios (proposed method). AUC evaluated over validation-period days; precision and recall computed at the z=3 threshold.

Scenario	Fault Type	AUC	Precision	Recall
SA +2 °C	Sensor	0.9992	1.000	0.833
SA +4 °C	Sensor	1.0000	1.000	0.983
SA −2 °C	Sensor	1.0000	1.000	1.000
SA −4 °C	Sensor	1.0000	1.000	1.000
Damper stuck 75%	Mechanical	1.0000	1.000	1.000
Valve stuck 50%	Mechanical	1.0000	1.000	1.000

**Table 7:** Fault isolation results for SD-AHU scenarios. The top-ranked sensor, its median daily anomaly score, isolation margin (rank-1 minus rank-2 score), and the top-3 ranking are reported. SA_TEMP ranks first for the ±4 °C biases; for the milder ±2 °C biases, MA_TEMP ranks first with SA_TEMP at rank 2.

Scenario	Top Sensor	Score	Margin	Top 3
Fault-free	SA_TEMP	−0.13	0.05	SA_TEMP, RA_TEMP, ZONE_TEMP_4
SA +2 °C	MA_TEMP	4.09	0.91	MA_TEMP, SA_TEMP, ZONE_TEMP_1
SA +4 °C	SA_TEMP	5.21	0.38	SA_TEMP, MA_TEMP, ZONE_TEMP_1
SA −2 °C	MA_TEMP	6.14	0.57	MA_TEMP, SA_TEMP, ZONE_TEMP_1
SA −4 °C	SA_TEMP	10.80	0.87	SA_TEMP, MA_TEMP, ZONE_TEMP_1
Damper stuck 10%	MA_TEMP	5.92	3.28	MA_TEMP, SA_TEMP, RA_TEMP
Damper stuck 25%	SA_TEMP	11.55	5.79	SA_TEMP, MA_TEMP, ZONE_TEMP_1
Damper stuck 75%	MA_TEMP	44.27	28.57	MA_TEMP, SA_TEMP, ZONE_TEMP_1
Valve stuck 10%	SA_TEMP	34.78	26.53	SA_TEMP, MA_TEMP, ZONE_TEMP_1
Valve stuck 50%	SA_TEMP	16.29	13.70	SA_TEMP, MA_TEMP, ZONE_TEMP_1
Valve stuck 75%	SA_TEMP	30.09	25.84	SA_TEMP, MA_TEMP, ZONE_TEMP_1
Valve leak	MA_TEMP	3.70	1.20	MA_TEMP, SA_TEMP, ZONE_TEMP_1

**Table 8:** Fault discrimination space coordinates. Sensor faults cluster at near-zero Damper_MAE and Valve_MAE; damper faults show elevated Damper_MAE only; valve faults show elevated Valve_MAE only. The three fault categories form non-overlapping clusters.

Scenario	Category	Anomaly Score	Damper MAE	Valve MAE
Fault-free	Fault-free	−0.12	0.0002	0.0008
SA +2 °C	Sensor	4.55	0.0000	0.0012
SA +4 °C	Sensor	6.93	0.0000	0.0017
SA −2 °C	Sensor	6.04	0.0000	0.0006
SA −4 °C	Sensor	9.93	0.0000	0.0005
Damper stuck 10%	Mechanical	4.09	0.346	0.0010
Damper stuck 25%	Mechanical	12.96	0.380	0.0010
Damper stuck 75%	Mechanical	12.81	0.581	0.0005
Valve stuck 10%	Mechanical	34.83	0.0000	0.640
Valve stuck 50%	Mechanical	13.54	0.0000	0.496
Valve stuck 75%	Mechanical	30.70	0.0000	0.750
Valve leak	Mechanical	3.35	0.0000	0.033

**Table 9:** AUC comparison across three detection methods on SD-AHU scenarios.

Scenario	Fault Type	Mixing Residual	PCA	Proposed
SA +2 °C	Sensor	0.4942	1.000	0.9992
SA +4 °C	Sensor	0.4906	1.000	1.000
SA −2 °C	Sensor	0.510	1.000	1.000
SA −4 °C	Sensor	0.5272	1.000	1.000
Damper stuck 10%	Mechanical	0.3639	1.000	1.000
Damper stuck 25%	Mechanical	0.7769	1.000	1.000
Damper stuck 75%	Mechanical	0.3319	1.000	1.000
Valve stuck 10%	Mechanical	0.5458	1.000	1.000
Valve stuck 50%	Mechanical	0.511	1.000	1.000
Valve stuck 75%	Mechanical	0.5000	1.000	1.000
Valve leak	Mechanical	0.5050	1.000	1.000

**Table 10:** FCU detection AUC: proposed virtual-sensor method versus PCA. The proposed method achieves AUC = 1.0 for all sensor bias scenarios; PCA achieves only 0.634–0.897 on the same scenarios.

Scenario	Fault Type	Proposed AUC	PCA AUC
RM +2 °C	Sensor	1.0000	0.6337
RM +4 °C	Sensor	1.0000	0.8887
RM −2 °C	Sensor	1.0000	0.6684
RM −4 °C	Sensor	1.0000	0.8966
Cooling valve stuck 50%	Mechanical	0.9786	1.0000
OA damper stuck 100%	Mechanical	0.9970	1.0000

**Table 11:** FCU fault isolation results. FCU_RAT is correctly identified as the primary anomalous sensor in all room temperature sensor bias scenarios. Mechanical fault isolation follows physical causality (cooling valve: FCU_CLG_RWT; heating valve: FCU_HTG_RWT; damper: FCU_MAT).

Scenario	Top Sensor	Score	Top 3
Fault-free	FCU_HTG_EWT	0.0	FCU_HTG_EWT, FCU_CLG_EWT, FCU_DAT
RM +2 °C	FCU_RAT	52.94	FCU_RAT, RM_TEMP, FCU_MAT
RM +4 °C	FCU_RAT	106.27	FCU_RAT, RM_TEMP, FCU_MAT
RM −2 °C	FCU_RAT	52.51	FCU_RAT, RM_TEMP, FCU_MAT
RM −4 °C	FCU_RAT	105.74	FCU_RAT, RM_TEMP, FCU_MAT
Cool valve stuck 50%	FCU_CLG_RWT	3.13	FCU_CLG_RWT, FCU_MAT, FCU_HTG_RWT
Cool valve stuck 100%	FCU_CLG_RWT	3.21	FCU_CLG_RWT, FCU_MAT, FCU_HTG_RWT
Heat valve stuck 50%	FCU_HTG_RWT	76.99	FCU_HTG_RWT, FCU_DAT, FCU_CLG_RWT
OA damper stuck 50%	FCU_MAT	1.71	FCU_MAT, FCU_CLG_RWT, FCU_CLG_EWT
OA damper stuck 100%	FCU_MAT	10.93	FCU_MAT, FCU_CLG_RWT, RM_TEMP

**Table 12:** Comparison of the proposed framework with the most relevant competing approaches. Only the proposed framework combines BRICK-driven automation, virtual sensor-based isolation, and confirmed cross-system validation (* validated on SD-AHU and FCU).

Method	Metadata-Driven	Virtual Sensors	Isolation	Cross-Sys.
Gao et al. 2024 [24]	Yes (Haystack)	No (Bayes. net)	Building-level	No
Hosamo et al. 2022 [26]	Yes (BRICK)	No (APAR)	Rule-based	No
Smagulova & Cerpa 2024 [25]	No	Physical sensors	Per-sensor	No
Gao et al. 2019 [18]	No	Yes (LSTM)	Per-sensor	No
Liu et al. 2021 [19]	No	Yes (Bayesian)	Per-component	No
Xu et al. 2025 [28]	No	No (PCA + LightGBM)	Per-sensor	No
Ren et al. 2023 [30]	No	No (deep learning)	No	Claimed
Yan et al. 2018 [11]	No	No (HMM + filter)	Per-sensor	No
Wang et al. 2010 [6]	No	No (PCA)	Per-sensor	No
Elnour et al. 2020 [23]	No	Yes (AANN)	Per-sensor	No
Yoon 2020 [20]	No	Yes (autoencoder)	Per-sensor	No
Choi & Yoon 2020 [21]	No	Yes (Gauss. proc.)	Per-sensor	
Papadopoulos et al. 2020 [16]	No	No (model-based)	Per-sensor	No
**Proposed**	**BRICK**	**LightGBM**	**Per-sensor**	Yes *

## Data Availability

The LBNL FDD benchmark dataset is publicly available at https://doi.org/10.25984/1881324. The BRICK schema ontology is available at https://brickschema.org/ (accessed on 23 May 2026). The analysis code is available from the first author upon request.
